# Numerical Simulation of the Fracture Behavior of High-Performance Fiber-Reinforced Concrete by Using a Cohesive Crack-Based Inverse Analysis

**DOI:** 10.3390/ma15010071

**Published:** 2021-12-23

**Authors:** Alejandro Enfedaque, Marcos G. Alberti, Jaime C. Gálvez, Pedro Cabanas

**Affiliations:** 1 Departamento de Ingeniería Civil, Construcción, E.T.S de Ingenieros de Caminos, Canales y Puertos, Universidad Politécnica de Madrid, 28040 Madrid, Spain; marcos.garcia@upm.es (M.G.A.); jaime.galvez@upm.es (J.C.G.); 2 Aerospace Department, Cranfield University, College Road, Cranfield, Wharley End, Bedford MK43 0AL, UK; pedro.cabanas.116@cranfield.ac.uk

**Keywords:** fiber-reinforced concrete, simulation, cohesive crack, fiber cocktail

## Abstract

Fiber-reinforced concrete (FRC) has become an alternative for structural applications due its outstanding mechanical properties. The appearance of new types of fibres and the fibre cocktails that can be configured by mixing them has created FRC that clearly exceeds the minimum mechanical properties required in the standards. Consequently, in order to take full advantage of the contribution of the fibres in construction projects, it is of interest to have constitutive models that simulate the behaviour of the materials. This study aimed to simulate the fracture behaviour of five types of FRC, three with steel fibres, one with a combination of two types of steel fibers, and one with a combination of polyolefin fibres and two types of steel fibres, by means of an inverse analysis based on the cohesive crack approach. The results of the numerical simulations defined the softening functions of each FRC formulation and have pointed out the synergies that are created through use of fibre cocktails. The information supplied can be of help to engineers in designing structures with high-performance FRC.

## 1. Introduction

Fiber-reinforced concrete (FRC) can be considered a composite material as it merges two materials seeking to improve the properties that they individually have. While the plain concrete matrix provides the stiffness and the compressive strength, the fibres enhance the reduced tensile strength and brittleness of plain concrete. The result of this combination is a material with a remarkable compressive strength and an improved flexural and tensile strength, ductility, toughness, and impact resistance among other characteristics. [1,2,3]. Several studies have pointed out that the improvement of properties that the fibres provide to FRC depend on several factors such as their modulus of elasticity, constituent material, strength, fiber geometry, orientation, and dosage, among others [4,5,6].

Among all the available types of fibres suitable for being added to concrete, steel fibres, when added to concrete forming steel fiber-reinforced concrete (SFRC), have been the most used ones in structural applications such as footbridges, pavements, or tunnel linings [7,8,9,10,11]. In these applications, steel fibres prevent crack propagation and brittle failure, providing concrete with an improved strength and ductility after cracking. Moreover, polymer fibres such as PVA or polyolefin ones have proven to be a suitable alternative for structural uses and also might have fewer durability issues in chemically hazardous environments such as marine ones [12,13,14]. Another possibility that has been recently explored is using combinations of fibres, the so-called fibre cocktails, in order to obtain high performance FRC with tailor made properties. Several authors have studied the mechanical properties of fibre cocktails of micro and macro steel fibres with outstanding results [15,16,17]. Fibre cocktails composed by polymer and steel fibres have been also studied, being already available in papers dealing with concrete formulations that combined polypropylene and steel fibres. In most cases, such mixture aimed at complementing the structural characteristics of steel fibres with reduced drying and plastic shrinkage or even improved fire resistance [18,19].

As was mentioned in the previous paragraph, outstanding FRC properties might be obtained by mixing different types of fibers, but, in all cases, if a structural application is sought, the material properties must comply with certain requirements set in standards. There are national standards and structural design recommendations that establish the minimum mechanical properties that a certain type of FRC should meet to take advantage of the contribution of the fibres to the material properties [20,21,22]. However, in order to fully exploit the mechanical properties that the fiber cocktails provide, it is also necessary to supply reliable constitutive models that can be used in the structural design.

Due to the difficulties of developing such models, different theories have tried to describe the fracture process of concrete and FRC. Among them, the crack band theory [23] assumes that cracking takes place in a band where the cracks are homogeneously distributed, with the behaviour of the material described by a stress-strain relationship. Other authors have employed fracture mechanics to obtain the crack tip intensity factor by combining the fracture toughness of plain concrete and the stress-crack relationship provided by the fibre contribution [24]. Another possibility was to idealise the cracked material as a discontinuous entity where the crack that is described by a stress-crack opening relationship divides two sides of undamaged material that behaves elastically [25]. This approach has been employed with successful results in various published research. For instance, it was shown to be adequate not only for reproducing the fracture behavior of ultra-high-performance fibre-reinforced concrete notched prismatic specimens of several sizes reinforced with steel fibres [26] but also to determine the fracture energy of SFRC notched cylindrical specimens [27]. Moreover, the use of the cohesive crack approach has been employed for separating the contribution of the fracture energy and the fibre bridging zone in FRC [28]. In this study it was revealed that layered FRC might be more efficient for obtaining optimised structural elements. As a common feature of all these studies, it has been observed that the key factor of the model was the softening function that relates the stress-crack width relationship of the material.

To obtain such a relationship, some authors employed a numerical inverse approach that identified the mechanical and fracture properties required for performing an advanced analysis [29]. Other authors performed an analytical inverse analysis [30]. In all cases it was shown that the stress-crack width relationship that defines the behaviour of the cracks that appear in FRC depends on several parameters such as the plain concrete mechanical properties, fibre type [30], fibre dosage [31], and specimen size [32], among others.

Following that rationale, this paper sought to numerically replicate the three-point bending fracture behaviour of the same plain concrete with 0.89% volume fraction of OL and 3D and 5D steel fibers, respectively, by using multi-linear softening functions (OL fibres are short, straight, high-strength steel fibres, 3D fibres are steel fibers with hooked ends, and 5D fibers are high-strength steel fibres with double-hooked ends). To achieve this goal, experimental data found in literature were employed. The volume fraction was chosen, according to [33], in order to manufacture high-performance fiber-reinforced concrete mixes able to broadly exceed the requirements set in [20,21,22]. To simulate the material behaviour, the suitability of using multilinear softening functions to represent the *σ-w* relation of the mentioned types of SFRC had to be determined. Moreover, once the previous tasks were fulfilled, the constitutive model of the same plain concrete with a binary and ternary cocktail of fibres was found. In addition, a discussion regarding the differences among the softening functions and their relationship with the fibre geometry was carried out.

The significance of this research relies on the potential optimization of the structural design that might be achieved by implementing the constitutive models in structural design software. Moreover, once the individual softening functions of each SFRC have been determined, the analytical deduction of the softening functions of the cocktails of fibres may be explored in future contributions. Another point that should be underlined is that the mentioned optimisation in the use of the material properties might result in remarkable reductions, or even a total substitution, of the steel-bar reinforcement without compromising the reliability of FRC structural elements. Besides, the reduction of the amount of steel used could suppose not only a decrease in the economic costs but also in an increment of the sustainability of the structure due to the savings in raw materials and safety and improvements of durability.

## 2. Material Production and Experimental Campaign

This section will briefly present the manufacturing process of the concrete formulations, the fracture behavior of the mixes was simulated in the following sections. A detailed description of the materials used and the steps taken can be seen in [33].

Five formulations of self-compacting FRC were prepared, adding several fiber combinations to the same plain concrete formulation. Such plain concrete was designed considering three aggregate types: two types of gravel with particle sizes between 4–8 mm and 4–12 mm, respectively, and one type of sand with particle sizes between 0–2 mm. The maximum size of the aggregates was 12.7 mm. In addition, limestone powder was employed, seeking to enhance the workability of the mixes. In all formulations a Portland cement-type EN 197-1 CEM I 52.5 was used as binder. A policarboxylic superplasticiser was added to the formulations pursuing to achieve the self-compactability of all types of concrete while in a fresh state. Tap water was also employed in concrete production. The proportions of all components can be seen in Table 1.

**Figure 1:**
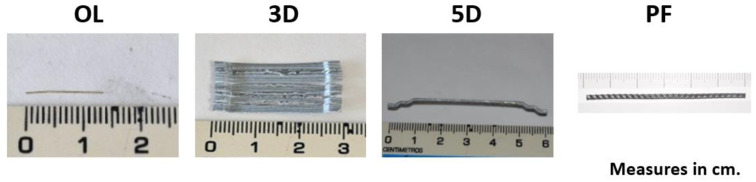
Appearance and length of the fibres used.

Three types of fibers (OL, 3D and 5D) were used in order to obtain three types of SFRC. Such formulations were termed SFRC-OL, SFRC-3D and SFRC-5D, respectively. In addition, two fibre cocktails were employed in the other two formulations. One of the hybrid formulations, which was named H1, was obtained by mixing OL and 5D fibres. The other hybrid formulation employed OL, 5D, and polyolefin fibers (PF). This last formulation was called H2. The fibers’ characteristics and their appearance can be seen in Table 2 and Figure 1, respectively.

The five concrete formulations were designed seeking to obtain high-performance concrete that was capable of surpassing the requirements set in the most common standards [20,21,22] for reducing or even eliminating the steel reinforcing bars. Consequently, the volume fraction of fibres added was equal to 0.89% in all concrete types. It should be pointed out that all the formulations were designed to meet the requirements that are set for self-compacting concrete while in a fresh state. As it was previously stated, three concrete types were SFRC with only one type of fibre and the other two were concrete types where a cocktail of fibres had been added. In H1 formulation, the volume fraction of OL and 5D fibres were equal to 0.445% and their sum was equal to 0.89%. Similarly, H2 had equal volume fractions of OL, 5D and PF, with each one being 0.293%, which is a volume fraction of 0.89% combined. It was observed that the experimental campaign had as a main target to characterize the mechanical behaviour of high-performance FRC and also analyse the synergies and improvements that could be obtained by combining several fibre types.

All concrete batches were manufactured in a planetary mixer with vertical axis following the same scheme. A thorough description of the process can be seen in [33]. From each batch, six cylindrical specimens of 150 mm of diameter and 300 mm of height were cast. In addition, four prismatic specimens of dimensions and 600 × 150 × 150 mm^3^ were also manufactured. All specimens were cured in a climatic chamber until 28 days of age. The cylindrical specimens were used to obtain the compressive strength, the modulus of elasticity, and the tensile strength following EN 12390-3 [34], EN 12390-13 [35], and EN 12390-6 [36], respectively. All tests were conducted at 28 days of age of the material. In Table 3, the results of the mentioned tests can be seen.

As can be seen in Table 3, there were no remarkable differences in the compressive strength of all the formulations tested. Likewise, no notable changes of the tensile strength were found among the mixes. On the contrary, regarding the modulus of elasticity, it should be highlighted that H1 boasted a modulus of elasticity that was clearly superior to the ones found in the other concrete types. However, for the purpose of this manuscript, such changes were of minor influence, as the modulus of elasticity only possessed a remarkable influence in the initial part of the fracture behaviour under a three-point bending setup.

**Figure 2:**
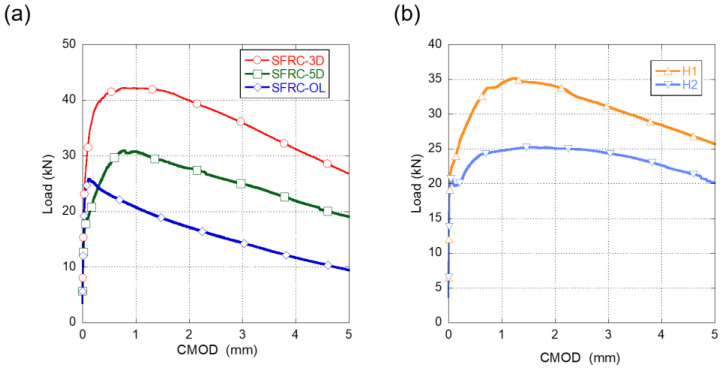
(**a**) Fracture test results of SFRC-OL, SFRC-3D, and SFRC-5D. (**b**) Fracture test results of H1 and H2.

The fracture behaviour was determined following the process shown in [37]. The results of the tests boasted a limited scattering, and the average of the results can be seen in Figure 2.

In Figure 2a it can be seen that, when the fiber type changed, the fracture behavior of the SFRC studied significantly varied. It can be seen that the overall best performance was obtained with the 3D fibres, followed by the 5D fibres and lastly by the OL fibres. SFRC-3D reached the highest load (maximum load and remaining load) followed by SFRC-5D and SFRC-OL. It is worth noting that, in SFRC-5D and SFRC-3D, alike, the maximum load appeared around 1 mm of CMOD, while in SFRC-OL, the maximum load was reached at a CMOD around 0.125 mm. After reaching the maximum load, all concrete specimens showed a progressive unloading.

The fracture behaviour of the concrete mixes where cocktail of fibres were added can be seen in Figure 2b.

In Figure 2a,b the curves shown correspond to at least the average behaviour of three specimens. It should be also highlighted that no remarkable scattering was found in the tests. Regarding the fracture behaviour of the mentioned formulations, it can be stated that the cocktails used in H1 and H2 clearly enhanced the fracture behaviour of any of the formulations that had only one type of fibres.

In order to determine if it is possible to consider the contribution of the fibres in the structural design and, consequently reduce the amount of steel reinforcement, [22] sets of the strength of the material at a CMOD value of 0.5 mm, which is often known as *f_R_*_1,_ should be at least 40% of the load at the limit of proportionality (*f_LOP_*). Moreover, it should be also checked that the load at a CMOD value of 2.5 mm should be at least 20% of *f_LOP_*, usually termed *f_R_*_3_. In Table 4, it can be observed that all concrete formulations exceeded the requirements set by [22].

Similarly to what was mentioned in the case of the production process, a detailed description and discussion of the results can be found in [33].

## 3. Numerical Simulations

The concept of the cohesive crack zone [25,38,39] was implemented in an element formulation, with an embedded cohesive crack, in order to reproduce the cracking process of the concrete formulations. Although this concept was firstly developed for plain concrete [40], it has been subsequently applied in fibre cementitious materials with successful results [41].

This approach considers that fracture takes place in mode I, and, hence, it was assumed that the cohesive stress vector **t** transmitted across the crack faces was parallel to the crack displacement vector **w** (central forces model). If the magnitude of the crack opening vector *|***w***|* does not decrease, the relation between both parameters can be stated as appears in (1)

(1) $$t=\frac{f\left(\left|w\right|\right)}{\left|w\right|}w\;\;\;\;\;\;$$

where *f*(|**w**|) is the softening function for pure opening, which, in this manuscript, will be defined by multilinear functions, where unloading and reloading branches are aligned with the origin and the softening function is defined by a set of points.

The mentioned formulation is referred to as constant strain triangular finite elements that only have one integration point. It should be mentioned that only the directions parallel to the triangle sides are considered as cracking directions.

If the element is cracked and, consequently, the crack direction is determined, the stress vector **t** can be obtained as

(2) $$t=\frac{A}{hL}\sigma \;n\;$$

It should be mentioned that the stress vector **t** is constant along the crack, with *h* being the triangle height, *A* representing the area of the element, *L* representing the crack length, **σ** representing the stress tensor, and **n** representing the unit vector normal to that side and to the crack. A more detailed explanation of the implementation can be found in [41].

In the cohesive crack theory, it is assumed that outside the crack the material remains elastic. The stress tensor can be obtained using Equation (3), where the inelastic behaviour is subtracted to correct the elastic deformation of the element by including the crack displacement.

(3) $$\sigma =E:\left[\varepsilon^{\alpha }-{\left(b^{+}⨂w\right)}^{S}\right]\;$$

In Equation (3), **E** stands for the elastic tangent tensor, *ε^α^* is the apparent strain vector obtained with the nodal displacements, and **b^+^** is the gradient vector corresponding to the shape function of the solitary node, as appears in Equation (4):

(4) $$b^{+}=\frac{1}{h}n\;$$

If it is considered that **t = σ n** and also using Equations (1) and (3), the following expression could be obtained.

(5) $$\left[\frac{f\left(\left|\tilde{w}\right|\right)}{\left|\tilde{w}\right|}1+\left[n\cdot E:b^{+}\right]\right]w=\left[E:\varepsilon^{\alpha }\right]\cdot n\;$$

where **1** stands for the identity tensor. If an iterative algorithm is used, **w** could be found to satisfy Equation (5).

The approach previously explained was introduced in a material subroutine in a commercial finite element code (ABAQUS) seeking to reproduce the material behaviour. An external file with the coordinates of the nodes and the elements is required to feed the subroutine with the geometry of the elements. If the material has not reached the tensile strength (*f_ct_*), the element behaves elastically. The tensile strength influences the limit of proportionality of the experimental curves, being greater as *f_ct_* increases. Nevertheless, if such value is exceeded, the direction of the maximum principal stress is found and a crack perpendicular to such direction is introduced. From that point onwards, the behaviour of the element is governed, if strains keep growing, by the softening function of the material, which relates the stress and the crack opening relation (*σ-w*). Apart from the tensile strength, the subroutine requires the modulus of elasticity of the material *E*. Although it is true that the presence of fibres in concrete changes the post-peak behaviour of the material when subjected to compressive stresses, given that for high crack widths the material behaviour is mainly under tensile stresses, no compressive damage was considered in the material implementation. Moreover, no experimental data appeared in [33] regarding the post-peak compressive strength of any of the mixes analysed.

The shape and characteristic points of the softening functions of all the mixes analysed were determined by the authors in the current study based on an iterative inverse analysis. The best reproduction of the fracture test results was sought. Such process can be summarised as the following steps. Firstly, the softening function is implemented in the element and the points that define it are set. Secondly, the simulation is performed and the behaviour of the numerical model when subjected to the fracture test is obtained. Thirdly, the experimental and the numerical behaviors are compared. This can be carried out using the load-deflection or the load-crack mouth opening displacement (CMOD) curves. If the numerical behavior does not reproduce the experimental behaviour, the points that define the softening function are modified and the next iteration starts again. If the numerical behaviour simulates with a certain degree of accuracy the experimental behavior, the points proposed in the first step are considered valid.

**Figure 3:**
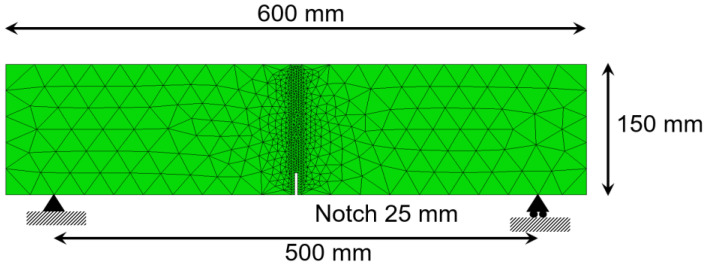
Mesh used in the numerical simulations.

In order to reproduce the material behavior, a plane mesh with plane stress conditions was employed. The mesh was composed of constant strain triangles, which are finer in the whereabouts of the notch. An image of one of the meshes can be seen in Figure 3. This scheme of mesh was considered valid in [32,42].

## 4. Results and Discussion

**Figure 4:**
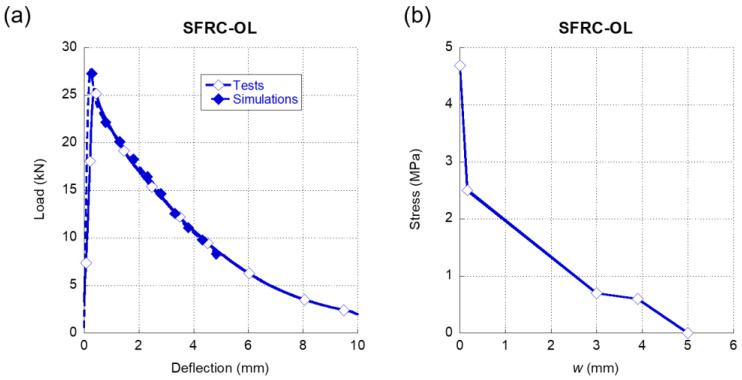
(**a**) Comparison of the average experimental results obtained in the fracture tests of SFRC-OL and the numerical simulation. (**b**) Softening function used in the simulation shown in (**a**).

In Figure 4a, the comparison of the results obtained in the experimental campaign and the simulations can be seen regarding SFRC-OL. As it can be clearly observed, the degree of accuracy that was achieved was remarkable, with the only slight difference present in the peak load. In contrast to what was shown in [41], the softening function in this occasion was composed by four straight stretches. The first one started when the opening of the crack was null and the stress was equal to the tensile strength registered in tests carried out in plain concrete. There was a sudden drop of the load-bearing capacity of the material in the initial stretch that might have been related to the crack width required by the fibres to contribute to the strength of SFRC-OL. Once the fibres started to enhance the mechanical properties of the concrete matrix, the load-bearing capacity decrease was more gradual. Such a feature was also perceived in the last two stretches of the softening function.

**Figure 5:**
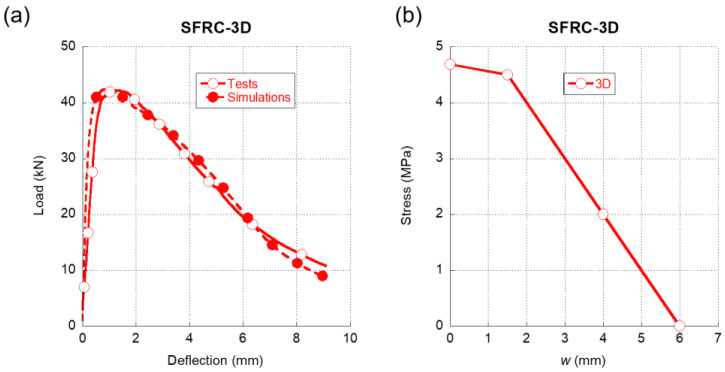
(**a**) Comparison of the average experimental results obtained in the fracture tests of SFRC-3D and the numerical simulation. (**b**) Softening function used in the simulation shown in (**a**).

Similarly to Figure 4, in Figure 5a we can see the comparison between the experimental results registered when testing SFRC-3D in a three-point bending fracture test and the results that were obtained in the numerical simulations. It should be highlighted that the accuracy of the simulation was remarkable not only in the peak load but also throughout the unloading branch of the test. Moreover, even at deflection values beyond 6 mm, the simulation was able to replicate the behaviour of SFRC-3D with precision. Regarding the softening function that enabled such accuracy, which is shown in Figure 5b, it should be underlined that, in contrast to what was shown in Figure 4b, there was no initial steep unloading for reduced crack widths. On the contrary, as can be perceived in Figure 5b, the initial part of the softening function maintained the load-bearing capacity close to the tensile strength of plain concrete. Such a feature might be related with the shape of the fibres that boasted hooked ends. Once this initial stretch was surpassed, there was a progressive loss in the load-bearing capacity of SFRC-3D, which is notoriously steeper than the first stretch.

**Figure 6:**
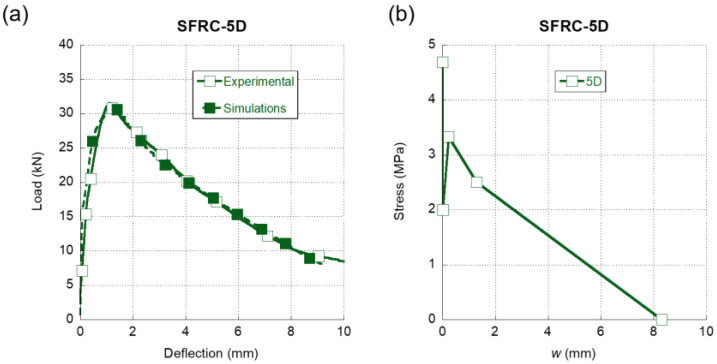
(**a**) Comparison of the average experimental results obtained in the fracture tests of SFRC-5D and the numerical simulation. (**b**) Softening function used in the simulation shown in (**a**).

In Figure 6a, we can observe the comparison between the experimental results of the three-point bending fracture tests of SFRC-5D and their numerical reproduction. The dashed line that represents the numerical simulation clearly follows the experimental trends with accuracy. The peak load was captured with precision and even the progressive loss of stiffness that the material suffered before reaching such point was reproduced. Moreover, there was an almost identical unloading in the simulation and in the experimental test from the peak load to the end of the test. Concerning the softening function that enabled such an accurate simulation, it should be mentioned that, in contrast with what was shown in Figure 5b, the softening function of SFRC-5D that can be seen in Figure 6b boasts a sudden decrease of the load-bearing capacity after surpassing the tensile strength. This phenomenon was not expected because such a load drop did not appear in the case of 3D fibres. If the crack width continued to grow, the load-bearing capacity would increase up to a local maximum, which would be followed by the final unloading of the material.

In this work, not only the experimental curves of the three-point bending fracture tests of the formulations with the addition of one fiber were simulated but also the formulations with an addition of cocktail of fibres. In the case of the formulation H1, the cocktail of fibres was composed by OL fibres and 5D fibres. At this point, it should be mentioned that the fracture curve was simulated following the same iterative process that was applied to the concrete formulation with only one type of fibre. Consequently, although the softening functions of SFRC-OL and SFRC-5D were known, the softening function of H1 could not be inferred from them.

**Figure 7:**
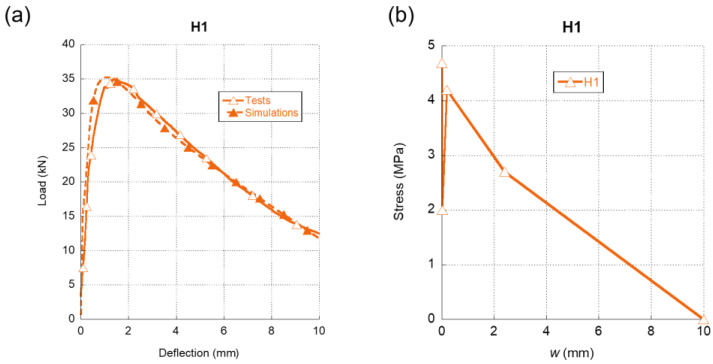
(**a**) Comparison of the average experimental results obtained in the fracture tests of H1 and the numerical simulation. (**b**) Softening function used in the simulation shown in (**a**).

In Figure 7a, we can see the comparison between the three-point bending fracture tests of H1 and the results obtained in the simulations. It can be observed that the simulations with the softening function, shown in Figure 7b, posed a high resemblance with the experimental results, both in the loading and unloading part of the curve. Regarding the softening function, it should be underlined that it featured the initial sudden load-bearing capacity drop typical of 5D fibres. Besides, the same steep recovery appeared right after the initial unloading took place. After reaching the maximum of this second stretch, there was a progressive unloading until the total collapse of the material. It should be also mentioned that the maximum crack width in the softening function of H1 was larger than any of the concrete formulations that had only one type of fibre.

**Figure 8:**
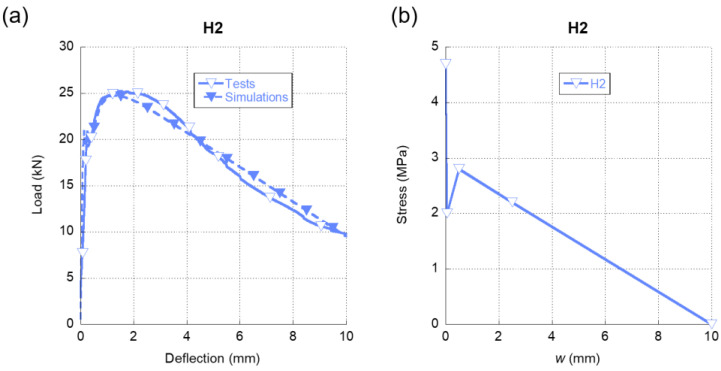
(**a**) Comparison of the average experimental results obtained in the fracture tests of H2 and the numerical simulation. (**b**) Softening function used in the simulation shown in (**a**).

Regarding the fracture behaviour of H2, in Figure 8a we can observe the precision obtained in the simulations if such results are compared with the experimental ones. It should be emphasised that the numerical simulations were able to reproduce not only the initial loading stretch but also the progressive loss of stiffness that the material suffered after reaching 20 kN until the peak load of the test was registered. It should be also mentioned that, as happened in the SFRC-5D and SFRC-3D tests, the peak load was registered gradually in contrast to the abrupt loss of stiffness observed in SFRC-OL. Such a feature entails the presence of 5D fibers capable of sustaining loads for reduced deflections. These two characteristics of the experimental curve were notably well captured by the numerical simulations. Moreover, a great similarity of the experimental and the numerical curves were seen during the complete test. If Figure 8b is analyzed, it can be mentioned that there was a sharp decrease of the load-bearing capacity of the material right after surpassing the tensile strength of plain concrete. Nevertheless, it can be also said that the presence of 5D and PF fibres enabled H2 to partially recover the load-bearing capacity even at low values of crack openings. Considering the notorious differences between the modulus of elasticity of 5D and PF fibres, 5D fibres might be considered the main responsible for such recovery. Another characteristic that should be underlined is that the maximum crack opening for this cocktail of fibres was remarkably greater than the ones shown in SFRC-3D, SFRC-5D, and SFRC-OL formulations.

The accuracy of the simulations performed in all the concrete mixes was assessed by comparing the fracture energies that were obtained in the experimental tests and the numerical simulations. In the last column of Table 5, the relative error of the fracture energy simulated can be seen, being in all cases below 2%.

## 5. Discussion

**Figure 9:**
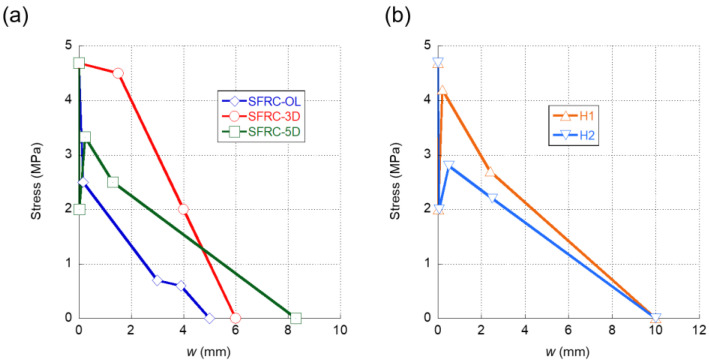
(**a**) Softening functions obtained in the inverse analysis of SFRC-OL, SFRC-3D, and SFRC-5D. (**b**) Softening functions obtained in the inverse analysis of H1 and H2.

In Figure 9a, a comparison of the softening functions of the SFRC formulations with only one type of fibre addition can be observed. It is obvious that there were significant differences among the three curves. The first feature that can be noted is that, in the case of the SFRC-OL and SFRC-5D formulations, right after surpassing the tensile strength, a sudden drop of the load bearing capacity of the material appeared. While this might be expected in the case of the SFRC-OL formulation due to the straight shape of the OL fibres, in the case of the 5D fibers, the double hook present in the ends of the fibres might have prevented any unloading from happening. Moreover, when seeking to determine the softening function of SFRC-5D, it became evident that the slight reduction of the stiffness that appeared in the load-deflection curves shown in Figure 6a was greatly influenced by this stretch of the softening function. In contrast to what was previously explained, the first stretch of the SFRC-3D softening function showed that the fibres could bear the load that the concrete matrix transferred to them when the tensile strength of the concrete was reached. Thus, such a stretch only showed a slight unloading.

After the first stretch, the softening function of the SFRC-OL fibers continued to develop a progressive unloading until the failure of the material took place. It should be mentioned that the slope of the rest of the unloading stages was much more progressive than the first stretch. This feature conferred SFRC-OL a certain ductility. Regarding the remaining part of the SFRC-3D softening function, it can be seen in Figure 9a that there was a load drop stretch until the collapse of the material happens. On the contrary, the softening function of SFRC-5D boasted an increment of the load-bearing capacity in the second stretch of the curve. Such an increment might be a result of the anchorage between the fibres and the concrete matrix. Once the anchorage fails, the material begins to unload until the failure is produced. Another feature that should be mentioned is that, in all concrete formulations, the maximum crack width was smaller than the length of the fibres added, with no evident relation to the fiber length. Such a relation was evident in the case of OL fibers, the length of which was 13 mm and the maximum crack width if which was 5 mm. Similarly, the length of 5D fibres was equal to 60 mm, while the maximum crack width was 8.3 mm. An analogue comment can be performed regarding 3D fibres.

In the case of the softening functions of H1 and H2 formulations, it can be observed in Figure 9b that they boasted similar shapes. Both of them had a rapid reduction of the load-bearing capacity right after reaching the tensile strength of the concrete matrix. This might be explained by the presence of 5D and OL fibres in both formulations. Moreover, in the case of H2, the presence of PF fibres in the fibre cocktail may have been also related with this feature. Regarding the re-loading stage of both softening functions, the H1 formulation was capable of gaining stress up to values close to the tensile strength of plain concrete. This feature might have been caused by the greater proportion of 5D fibres present in the H1 mix. On the contrary, H2 boasted a re-loading stage more subtle and with less slope than the corresponding stretch of H1. As was stated in [43], the softening functions that can represent the fracture behavior of polyolefin fibre-reinforced concrete (PFRC) mixes boasted a reloading stage after the first unloading. Therefore, such changes might have accounted for the presence of a greater number of 5D fibres in H1 and also for the lower modulus of elasticity of the PF fibres present in H2. Another feature that should be underlined is that the softening functions of H1 and H2 alike had maximum crack widths that were greater than any of the softening functions of the concretes, where only one type of fiber was added. This increment of the maximum crack width might be an effect of the synergies that appear when cocktails of fibres are used.

A comparison among the points that define the softening functions of the five mixes analysed can be seen in Table 6. The mentioned features can be clearly perceived comparing the coordinates of the points that define the softening functions.

## 6. Conclusions

This study implemented multi-linear softening functions in a user material subroutine of a finite element code (ABAQUS) in order to simulate the three-point bending fracture behaviour of three SFRC and two FRC with cocktails of fibres. The results obtained showed that the methodology used is a robust and efficient approach to simulate the fracture behaviour of the concrete mixes analysed.

The mentioned approach reproduced the fracture behaviour of the five concrete types studied with accuracy, obtaining less than a 2% difference between the experimental and the simulated fracture energy.

The analysis of the softening function corresponding to SFRC-5D showed that the presence of the double-hooked end in the 5D fibres might not provide an increment of the fiber–matrix anchorage for reduced CMOD values. However, in the case of 3D fibers, no load drops appeared at initial crack widths of the softening function. Regarding SFRC-OL, its softening function boasted a progressive unloading of the material. Lastly, no relationship was observed between the length of the fibres and the maximum crack width of the softening functions found.

Regarding the softening functions of H1 and H2, the presence of OL and 5D fibres in both cocktails explains the presence of an unloading stretch and a re-loading stage. The synergies that appeared when combining several types of fibres generated an increment of the maximum crack width both in H1 and H2. However, a further analysis should be performed for a better measurement of the enhancement that the combination of fibers can provide.

## Figures and Tables

**Table 1:** Plain concrete formulation.

Component	Weight (kg/m^3^)
Water	199
Cement	425
Limestone filler	210
Sand	947
Gravel	292
Finer gravel	194
Superplasticizer	5.91

**Table 2:** Fibre characteristics.

	OL	3D	5D	PF
Material	Steel	Steel	Steel	Polyolefin
Shape	Straight	Hooked	Double-hooked	Straight
Length (mm)	13	30	60	60
Eq. diameter (mm)	0.2	0.38	0.9	0.9
Tensile strength (MPa)	>2600	>1160	>2300	>500
Modulus of elasticity (GPa)	210	210	210	9
Fibres per kg.	282,556	3183	3132	27,000

**Table 3:** Mechanical properties and their corresponding coefficient of variation (c.v.) of the concrete mixes [33].

Formulation	*f* *_cm_*		*E*		*f* *_ct_*	
	(MPa)	c.v.	(GPa)	c.v.	(MPa)	c.v.
SFRC-OL	66.9	0.05	32.8	0.01	7.15	0.03
SFRC-3D	63.1	0.04	32.7	0.02	7.96	0.04
SFRC-5D	63.8	0.02	33.9	0.02	7.69	0.09
H1	64.7	0.02	40.7	0.03	8.05	0.01
H2	66.3	0.03	29.5	0.01	7.95	0.01

**Table 4:** Residual strength of the concrete formulation in relation with the requirements set by [22].

Strength (MPa)	*f_LOP_*	*f_R_*_1_ (0.5 mm)	*% f_LOP_*	*f_R_*_3_ (2.5 mm)	*% f_LOP_*
SFRC-OL	7.3	7.39	102%	5.03	69%
SFRC-5D	5.5	8.11	148%	8.40	153%
SFRC-3D	8.2	13.18	161%	12.17	148%
H1	7.0	9.94	142%	10.33	147%
H2	6.6	7.51	113%	7.97	120%

**Table 5:** Comparison of the experimental and simulated fracture energies at a deflection of 10 mm.

Concrete Mix	Experimental *W_f_* (kN/mm)	Simulation *Wf* (kN/mm)	Experimental *G_f_* (N/m)	Simulation *G_f_* (N/m)	Δ*G_f_* (%)
SFRC-OL	98.8	97.1	5270.4	5179.7	−1.75%
SFRC-3D	236.8	241.5	12,627.8	12,878.1	1.94%
SFRC-5D	170.5	172.8	9095.8	9213.9	1.28%
H1	232.4	235.2	12,396.2	12,546.1	1.20%
H2	178.9	181.1	9542.9	9657.4	1.19%

**Table 6:** Softening functions of the concrete mixes analysed.

Concrete Mix	SFRC-OL		SFRC-3D		SFRC-5D		H1		H2	
	w (mm)	σ (MPa)	w (mm)	σ (MPa)	w (mm)	σ (MPa)	w (mm)	σ (MPa)	w (mm)	σ (MPa)
Point 1	0.00	4.69	0.00	4.69	0.00	4.69	0.00	4.69	0.00	4.69
Point 2	0.16	2.50	1.50	4.50	0.02	2.00	0.02	2.00	0.05	2.00
Point 3	3.00	0.70	4.00	2.00	0.23	3.33	0.20	4.20	0.50	2.80
Point 4	3.90	0.60	6.00	0.00	1.30	2.50	2.40	2.70	2.50	2.20
Point 5	5.00	0.00	-	-	8.30	0.00	10.00	0.00	10.00	0.00

## Data Availability

The data presented in this study are available on request from the corresponding author.

## References

[1] Brandt A. (2008). Fibre reinforced cement-based (FRC) composites after over 40 years of development in building and civil engineering. Compos. Struct..

[2] Naaman E. (2003). Engineered steel fibers with optimal properties for reinforcement of cement composites. J. Adv. Concr. Technol..

[3] Zollo R. (1996). Fiber-reinforced concrete: An overview after 30 years of development. Cem. Concr. Compos..

[4] Pająk M., Ponikiewski T. (2013). Flexural behavior of self-compacting concrete reinforced with different types of steel fibers. Constr. Build. Mater..

[5] Giaccio G., Tobes J.M., Zerbino R. (2008). Use of small beams to obtain design parameters of fibre reinforced concrete. Cem. Concr. Compos..

[6] Enfedaque A., Alberti M.G., Gálvez J.C. (2019). Influence of fiber distribution and orientation in the fracture behavior of polyolefin fiber-reinforced concrete. Materials.

[7] Lopez J.A., Serna P., Camacho E., Coll H., Navarro-Gregori J. (2014). First ultra-high-performance fibre-reinforced concrete footbridge in Spain: Design and construction. Struct. Eng. Int..

[8] Jafarifar N., Pilakoutas K., Bennett T. (2016). The effect of shrinkage cracks on the load bearing capacity of steel-fibre-reinforced roller-compacted-concrete pavements. Mater. Struct..

[9] De la Fuente A., Pujadas P., Blanco A., Aguado A. (2012). Experiences in Barcelona with the use of fibres in segmental linings. Tunn. Undergr. Space Technol..

[10] Yu T., Remennikov A.M. (2014). Novel hybrid FRP tubular columns for sustainable mining infrastructure: Recent research at University of Wollongong. Int. J. Min. Sci. Technol..

[11] Liu P., Zhou X., Qian Q. (2021). Experimental investigation of rigid confinement effects of radial strain on dynamic mechanical properties and failure modes of concrete. Int. J. Min. Sci. Technol..

[12] Alberti M.G., Enfedaque A., Gálvez J.C., Pinillos L. (2017). Structural Cast-in-Place Application of Polyolefin Fiber–Reinforced Concrete in a Water Pipeline Supporting Elements. J. Pipeline Syst. Eng..

[13] Alberti M.G., Enfedaque A., Gálvez J.C. (2015). Improving the Reinforcement of Polyolefin Fiber Reinforced Concrete for Infrastructure Applications. Fibers.

[14] Hossain K.M.A., Lachemi M., Sammour M., Sonebi M. (2013). Strength and fracture energy characteristics of self-consolidating concrete incorporating polyvinyl alcohol, steel and hybrid fibres. Constr. Build. Mater..

[15] Okeh C.A., Begg D.W., Barnett S.J., Nanos N. (2019). Behaviour of hybrid steel fibre reinforced self-compacting concrete using innovative hooked-end steel fibres under tensile stress. Constr. Build. Mater..

[16] Caggiano A., Cremona M., Faella C., Lima C., Martinelli E. (2012). Fracture behavior of concrete beams reinforced with mixed long/short steel fibers. Constr. Build. Mater..

[17] Rambo D.A.S., de Andrade Silva F., Toledo Filho R.D. (2014). Effect of steel fiber hybridization on the fracture behavior of self-consolidating concretes. Cem. Concr. Compos..

[18] Caetano H., Rodrigues J.P.C., Pimienta P. (2019). Flexural strength at high temperatures of a high strength steel and polypropylene fibre concrete. Constr. Build. Mater..

[19] Sivakumar A., Santhanam M. (2007). A quantitative study on the plastic shrinkage cracking in high strength hybrid fibre reinforced concrete. Cem. Concr. Compos..

[20] EHE-08, Spanish Structural Concrete Code, Spanish Minister of Public Works, 2008. https://www.mitma.gob.es/recursos_mfom/contents.pdf.

[21] CNR-DT 204, Guide for the Design and Construction of Fiber-Reinforced Concrete Structures, Consiglio Nazionale Delle Riserche, Roma, 2006. http://hdl.handle.net/11386/2293864.

[22] (2010). MC2010, Fib Model Code.

[23] Bazant Z.P., Oh B.H. (1983). Crack band theory for fracture of concrete. Mater. Struct..

[24] Zhang J., Li V.C. (2004). Simulation of crack propagation in fiber-reinforced concrete by fracture mechanics. Cem. Concr. Res..

[25] Hillerborg A., Modéer M., Petersson P.E. (1976). Analysis of crack formation and crack growth in concrete by means of fracture mechanics and finite elements. Cem. Concr. Res..

[26] Awinda K., Chen J., Barnett S., Fox D. (2014). Modelling behaviour of ultra high performance fibre reinforced concrete. Adv. Appl. Ceram..

[27] Kazemi M.T., Fazileh F., Ebrahiminezhad M.A. (2007). Cohesive crack model and fracture energy of steel-fiber-reinforced-concrete notched cylindrical specimens. J. Mater. Civ. Eng..

[28] Park K., Paulino G.H., Roesler J. (2010). Cohesive fracture model for functionally graded fiber reinforced concrete. Cem. Concr. Res..

[29] Sucharda O., Pajak M., Ponikiewski T., Konecny P. (2017). Identification of mechanical and fracture properties of self-compacting concrete beams with different types of steel fibres using inverse analysis. Constr. Build. Mater..

[30] Gao D., Ding C., Pang Y., Chen G. (2021). An inverse analysis method for multi-linear tensile stress-crack opening relationship of 3D/4D/5D steel fiber reinforced concrete. Constr. Build. Mater..

[31] Enfedaque A., Alberti M.G., Gálvez J.C. (2019). Analysis of the Versatility of Multi-Linear Softening Functions Applied in the Simulation of Fracture Behaviour of Fibre-Reinforced Cementitious Materials. Materials.

[32] Suárez F., Gálvez J.C., Alberti M.G., Enfedaque A. (2021). Fracture and size effect of PFRC specimens simulated by using a trilinear softening diagram: A predictive approach. Materials.

[33] Alberti M.G., Enfedaque A., Gálvez J.C., Cortez A. (2020). Optimisation of fibre reinforcement with a combination strategy and through the use of self-compacting concrete. Constr. Build. Mater..

[34] EN 12390-3, Testing Hardened Concrete. Part 3: Compressive Strength of Test Specimens, 2009. https://www.en-standard.eu/bs-en-12390-3-2019-testing-hardened-concrete-compressive-strength-of-test-specimens/.

[35] EN 12390-13, Testing Hardened Concrete—Part 13: Determination of Secant Modulus of Elasticity in Compression, 2013. https://www.en-standard.eu/csn-en-12390-13-testing-hardened-concrete-part-13-determination-of-secant-modulus-of-elasticity-in-compression-4/.

[36] EN 12390-6, Testing Hardened Concrete. Part 6. Tensile Splitting Strength of Test Specimens, 2009. https://www.en-standard.eu/bs-en-12390-6-2009-testing-hardened-concrete-tensile-splitting-strength-of-test-specimens/.

[37] EN 14651:2005+A1, Test Method for Metallic Fibre Concrete. Measuring the Flexural Tensile Strength (Limit of Proportion-Ality (LOP), Residual), 2007. https://www.en-standard.eu/bs-en-14651-2005-a1-2007-test-method-for-metallic-fibre-concrete-measuring-the-flexural-tensile-strength-limit-of-proportionality-lop-residual/.

[38] Barenblatt G.I. (1962). The mathematical theory of equilibrium cracks in brittle fracture. Advances in Applied Mechanics.

[39] Dugdale D.S. (1960). Yielding of steel sheets containing slits. J. Mech. Phys. Solids..

[40] Guinea G.V., Planas J., Elices M. (1994). A general bilinear fit for the softening curve of concrete. Mater. Struct..

[41] Enfedaque A., Alberti M.G., Gálvez J., Domingo J. (2017). Numerical simulation of the fracture behaviour of glass fibre reinforced cement. Constr. Build. Mater..

[42] Suárez F., Gálvez J.C., Enfedaque A., Alberti M.G. (2019). Modelling fracture on polyolefin fibre reinforced concrete specimens subjected to mixed-mode loading. Eng. Fract. Mech..

[43] Alberti M.G., Enfedaque A., Gálvez J.C., Reyes E. (2017). Numerical modelling of the fracture of polyolefin fibre reinforced concrete by using a cohesive fracture approach. Compos. Part B Eng..

